# *Rickettsia parkeri* genetic diversity from three different hard tick species (family: Ixodidae)

**DOI:** 10.1186/s13071-026-07276-6

**Published:** 2026-02-26

**Authors:** Madeleine Meyer Torelli, Lídia Gual-Gonzalez, Kristy Baus, Melissa S. Nolan

**Affiliations:** 1 Department of Epidemiology and Biostatistics, Arnold School of Public Health, University of South Carolina, Columbia, SC 29208 USA; 2 Institute for Infectious Disease Translational Research, University of South Carolina, Columbia, SC 29208 USA; 3 Department of Public Health Sciences, College of Behavioral, Social and Health Sciences, Clemson University, Clemson, SC 29634 USA

**Keywords:** *Rickettsia parkeri*, Arachnid vectors, Lone star tick, *Amblyomma americanum*, Spotted fever group *Rickettsia*, Nanopore sequencing, Long read sequencing

## Abstract

**Background:**

*Rickettsia parkeri* sensu stricto, a causative agent of spotted fever rickettsiosis, is spread via the bite of infected ticks within the Amblyomma maculatum complex group. In the United States of America (USA), Am. maculatum Koch, 1844 is the primary vector for R. parkeri; however, Amblyomma americanum (Linnaeus, 1758) and Dermacentor variabilis (Say, 1821) have demonstrated potential to transmit R. parkeri under laboratory conditions. In this study, we investigate the genetic differences between R. parkeri detected in Am. maculatum, the primary enzootic vector, and potential secondary vectors – Am. americanum and De. variabilis.

**Methods:**

Using Nanopore long-read amplicon sequencing, we compared four R. parkeri genes amplified from naturally infected *Am. maculatum, Am. americanum*, and De. variabilis collected in the USA. Three R. parkeri genes associated with potential virulence factors were sequenced: outer membrane protein A (OmpA/sca0), outer membrane protein B (OmpB/sca5), and surface cell antigen 4 (*gene D/sca4*). One species-level gene target was used for species confirmation: 16S ribosomal RNA gene (16S).

**Results:**

Differences in cellular-entry and pathogen chromosomal genes (OmpA, OmpB, and 16S) were detected within the different tick species. No differences were noted in the cell-to-cell mediated transfer gene (*gene D*) between tick species.

**Conclusions:**

This preliminary study suggests that infection in Am. americanum may lead to changes in R. parkeri genes responsible for pathogen-host cell attachment and replication processes, but once established in a host cell, R. parkeri transfer between cells is unlikely to be impacted.

**Supplementary Information:**

The online version contains supplementary material available at 10.1186/s13071-026-07276-6.

## Background

*Rickettsia parkeri* is an obligate intracellular bacterium classified within the spotted fever group *Rickettsia* (SFGR) [1]. Spotted fever rickettsioses are mild to life-threatening illnesses caused by the bite of an infected hard tick (Acari: Ixodidae), and often, there is a conserved species-specific SFGR and tick association [2, 3]. In the case of *Rickettsia parkeri* sensu stricto (s.s.), the *Amblyomma maculatum* complex ticks are considered primary bacterium vectors [2–6]. Three separate *R. parkeri* strains have been identified in South America—the Atlantic Rainforest strain (vector: *Amblyomma ovale*), the NOD strain (vector: *Ambloymma nodosum*), and the Parvitarsum strain (vector: *Ambloymma parvitarsum*) [6–8]. Despite *R. parkeri* s.s. being associated with *Am. maculatum* in the USA, *R. parkeri* has been found naturally infecting other local tick species [7, 9–11]. Our previous work has confirmed these results [12], leading us to question whether *R. parkeri* infection outside its primary enzootic vector might place selective pressure on *R. parkeri* genes.

Research on the molecular mechanisms driving *R. parkeri* infection within vectors is scarce, yet evidence shows that the same genes driving pathogenicity in mammalian cells may also play a role in tick infection [13, 14]. The rickettsial outer membrane protein B (OmpB) contributes to *R. parkeri* s.s. infectivity in *Am. maculatum* ticks [1, 13]. This protein is also associated with mammalian infection, as it pairs with a second outer membrane protein, OmpA, in driving the initial bacterial attachment and cell invasion process [1]. Surface cell antigen 4 (*sca4*), also known as “*gene D*” is responsible for interrupting vinculin and α-catenin binding in cells, which promotes cell-to-cell transportation of *R. parkeri* [15, 16] (see Graphical Abstract).

To assess whether *R. parkeri* bacteria detected in wild primary and secondary vectors exhibited differences in four key genes, we conducted Nanopore long-read amplicon sequencing on three different species of naturally infected Ixodidae collected from South Carolina and Virginia, USA.

## Methods

The *R. parkeri* genetic pathogen sequences isolated from 79 tick samples were assessed to address our study objectives: *Am. maculatum* (*n* = 7), *Am. americanum* (*n* = 54), and *Dermacentor variabilis* (*n* = 18). Tick samples were sourced from previous surveillance projects, and included pooled, flat, questing ticks collected from South Carolina and Virginia, and individual host-attached ticks collected from South Carolina animal shelters. Available samples were limited by collection yields, so equal sample numbers for each tick species and collection source were not possible. All selected samples had previously tested *R. parkeri*-positive from existing surveillance studies using real time polymerase chain reaction testing per the US Centers for Disease Control’s (CDC) SFGR pathogen testing protocol [17]. Ticks were morphologically identified to species using multiple dichotomous keys [18–24]. All *Am. maculatum* and *De. variabilis* ticks were host-attached (ranging from flat to engorged) and sourced from South Carolina animal shelters. All *Am. americanum* ticks were pooled questing ticks from Virginia and South Carolina. A positive control of *R. parkeri* s.s. strain NIAID Maculatum-20 DNA was provided by the CDC and used for quality control and comparison. Amplicon Nanopore sequencing was conducted targeting four *R. parkeri* genes: 16S ribosomal RNA (16S), *OmpA*, *OmpB*, and surface cell antigen 4 (*gene D/sca4*). A detailed description of the methods and associated literature references are provided in Supplementary Materials.

**Fig. 1:**
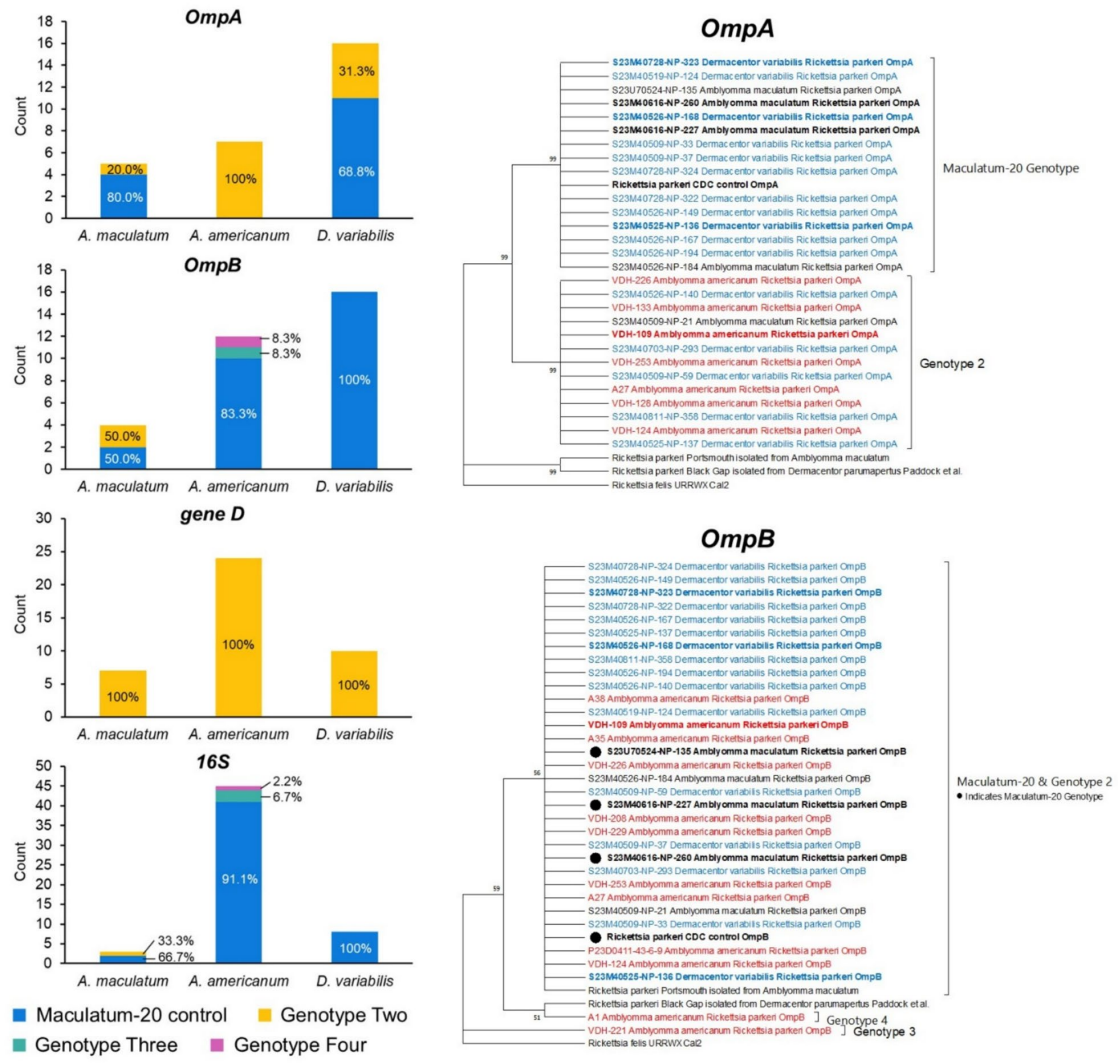
(*Left*) Percent of *R. parkeri* genotype presence grouped by originating tick species, where the vertical axis denotes the number of tick samples (pooled or individual) per species analyzed. (*Right*) *OmpA* and *OmpB* phylogenetic trees with labeled genotypes. Leaves in bold type face indicate samples in the final concatenated tree (Fig. 2). *R. parkeri* sequences from *Amblyomma americanum* tick pools are labeled in red, *Dermacentor variabilis* are labeled in blue, and *Amblyomma maculatum* are labeled in black. Accession numbers for the Portsmouth Black Gap strains are CP003341 and KY12456–KY124560, respectively. *Rickettsia felis* outgroup sequences are from accession number CP000053.1. All consensus sequences generated via this study have been published to GenBank with their accession numbers listed in Supplement 3.

**Fig. 2:**
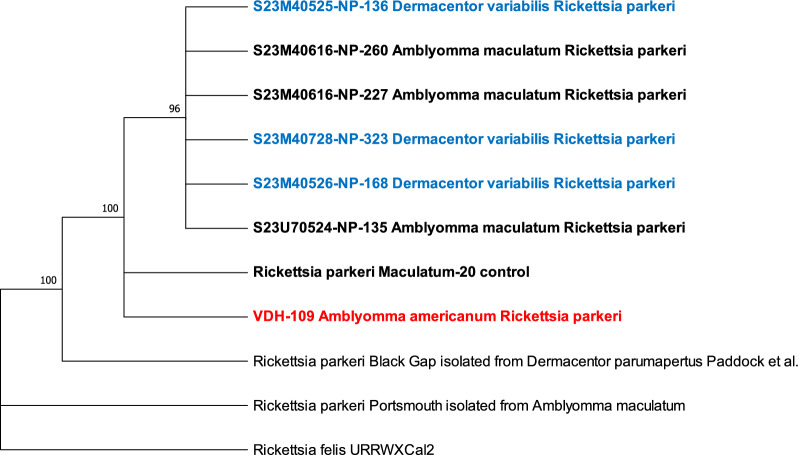
Maximum likelihood tree generated using potential *R. parkeri* virulence genes: *OmpA*, *OmpB*, and *gene D*. Leaves in bold indicate samples sequenced in this experiment. Accession numbers for the Portsmouth and Black Gap strains are CP003341 and KY12456–KY124560, respectively. *Rickettsia felis* outgroup sequences are from accession number CP000053.1. All consensus sequences generated via this study have been published to GenBank with their accession numbers listed in Supplement 3.

Tick samples that yielded *R. parkeri* consensus sequences for the three potential virulence genes were concatenated in the expected gene order. Two *R. parkeri* strains were found on GenBank with sequences for the same three gene targets. To allow for tree rooting, *Rickettsia felis* was used as an outgroup with the gene target sequences of interest extracted from GenBank (accession number CP000053.1) [7]. GenBank targets were similarly concatenated and then aligned to our sample results using MUSCLE [25]. A maximum likelihood tree was generated in MEGA11 using a bootstrap method with 500 replicates [26]. Complete evolutionary analysis methods are provided in Supplement 1. The resulting phylogenetic trees are provided in Figs. 1 and 2. Branch lengths are arbitrary and do not indicate any relationship in this tree. Numbers next to branches within the tree indicate the percentage of replicate trees in which the associated taxa are clustered together [27].

## Results

Two genotypes were described for *OmpA*: one corresponded with the Maculatum-20 control, and the second contained eight nucleotide substitutions corresponding to five amino acid changes. Four genotypes were observed for *OmpB*: the Maculatum-20 control, a genotype containing a single nucleotide deletion at locus 338, a third genotype with 27 nucleotide substitutions, and finally a fourth genotype with 22 nucleotide substitutions. The second genotype for *OmpB* resulted in a reading frameshift. The third genotype corresponded to dramatic differences in protein translation compared with the control genotype. An NCBI Basic Local Alignment Search Tool (BLAST) search indicated that the amplicon aligned best with *R. amblyommatis*. Despite the substitutions, the fourth and final genotype still yielded *R. parkeri* BLAST results but translated to a 17 amino acid difference in the protein sequence. Two genotypes were observed for *gene D*: the Maculatum-20 control and one containing 23 nucleotide substitutions which corresponded to 15 amino acid substitutions compared with the control genotype. Finally, four genotypes were observed for 16S: the Maculatum-20 control, a single nucleotide substitution at locus 728, a genotype with a 59 bp deletion at the start of the amplicon, and one containing 25 nucleotide substitutions throughout the amplicon with a 5 bp insertion at loci 390–395. Percent genotype distributions by tick species are graphically depicted in Fig. 1. Genotype distribution by geographic location (i.e. Virginia or South Carolina) is provided in Supplementary Fig. 2.

*Rickettsia parkeri* sequences from *De. variabilis* and *Am. maculatum* ticks were more genetically similar than those pathogen sequences isolated from *Am. americanum* ticks (Fig. 2). *Rickettsia amblyommatis* co-infections among *Am. americanum* ticks were pervasive, which limited the number of samples that could be included in the phylogenetic tree (Table 1). Specifically, 87% of *OmpA* and 78% of *OmpB R. parkeri* amplifications in *Am. americanum* ticks yielded *R. amblyommatis* consensus sequences, requiring those samples to be excluded from the phylogenetic tree. *gene D* amplification targets were more specific for *R. parkeri* (only 30% of gene D sequences yielded *R. amblyommatis* consensus sequences), improving the architecture of the phylogenetic tree.

**Table 1:** The number of tick samples of each species tested and the number of *R. parkeri* gene targets successfully amplified and sequenced for each species

Species	*OmpA* (*n* = 28)	*OmpB* (*n* = 33)	*gene D* (*n* = 51)	*16S* (*n* = 56)	All genes (*n* = 7)
*Amblyomma maculatum* 8.9% (*n* = 7)	5	5	7	3	3
*Amblyomma americanum* 68.4% (*n* = 54)	7	12	34	45	1
*Dermacentor variabilis* 22.8% (*n* = 18)	16	16	10	8	3

## Discussion

This preliminary analysis yielded interesting differences in *R. parkeri OmpA* and *OmpB* genes. For *OmpA*, the majority of *Am. maculatum* and *De. variabilis* contained *R. parkeri* sequences matching the Maculatum-20 strain, while all *Am. americanum* samples yielded a different genotype. For *OmpB*, all tick species were predominantly aligned with the Maculatum-20 strain, while both *Am. maculatum* and *Am. americanum* had alternative genotypes. It is worth noting that while four genotypes were observed from the raw sequencing data, the Maculatum-20 and genotype 2 sequences clustered together in the *OmpB* phylogenetic tree, implying that the bootstrap values were not robust enough to confidently separate the sequences into two genotypic groups. This is particularly interesting as the single nucleotide polymorphism (SNP) difference between the two observed sequence moieties resulted in a frame shift for genotype 2, which might indicate functional differences between the two protein expressions. These results provide preliminary evidence that host tick species might place selective pressure on *R. parkeri* outer membrane protein genes as a consequence of the bacterium’s establishment of infection in secondary vectors, with *OmpA* representing the gene with the strongest host species difference. As *gene D* is associated with the cell-to-cell movement of *R. parkeri*, the presence of a single genotype among *gene D* sequences, regardless of tick species, is interesting and implies that once intracellular infection is established, cell-to-cell propagation may not be impacted by tick host species [15, 16].

This paper had some limitations worth noting. First, as noted in the phylogenetic tree, both the *Am. maculatum* and *De. variabilis*/*R. parkeri* sequences grouped together in a separate clade branching off from the Maculatum-20 control, while the *Am. americanum* sample was genetically similar to the control. It is worth noting that this *Am. americanum* sample was from a pool of questing ticks collected in Virginia (USA), while the other ticks were individual ticks collected from animal shelters in South Carolina (USA). In addition, animal shelter ticks might have had freshly ingested blood, challenging the ability to distinguish between genetic variability derived from mammalian-host driven selective pressure versus true tick-driven variability. Simply put, in this analysis one cannot distinguish between freshly ingested *R. parkeri* from bloodmeal and a true, established infection within the tick. The sample size differences among the three tick species, as well as the *R. amblyommatis* presence in a number of *Am. americanum* leading to only one sample suitable for phylogenetic analysis, were additional study limitations. Care should be taken when interpreting these results as future work is needed to confirm that the phylogenetic differences observed are truly influenced by infection within the tick species and not simply random nonadaptive mutations clustered by geographic location or infectious blood meal status.

## Conclusions

Overall, this pilot study provided preliminary evidence that natural infection in *Am. americanum* may be causing selective pressure on *R. parkeri* genes, particularly that of the outer membrane protein A. The study’s natural collection methods limited sample size and inhibited infection timing inference so further controlled lab experiments are necessary. Finally, some ticks were pooled, preventing individual tick-level information. Despite these limitations, these results represent novel findings of interest to the scientific community, and we hope encourages further research aimed at understanding the potential relationship between genetic polymorphisms and secondary vector infection.

## Supplementary Information

Additional file 1

## Data Availability

Data is provided within the manuscript or supplementary information files.

## Abbreviations

BLAST: Basic Local Alignment Search Tool
CDC: Centers for Disease Control and Prevention
NCBI: National Center for Biotechnology Information
NIAID: National Institute of Allergy and Infectious Diseases
OmpA: Outer membrane protein A
OmpB: Outer membrane protein B
sca0: Surface cell antigen 0
sca4: Surface cell antigen 4
sca5: Surface cell antigen 5
s.s.: Sensu stricto
SFGR: Spotted fever group *Rickettsia*
USA: United States of America
