# Supplementary material for: Rickettsia parkeri genetic diversity from three different hard tick species (family: Ixodidae)
Source: Parasit Vectors. 2026 Feb 26;19:249. doi: 10.1186/s13071-026-07276-6 (PMC13267649; doi:10.1186/s13071-026-07276-6)
Supplement: Supplementary file 1 — Additional file 1 [file 13071_2026_7276_MOESM1_ESM.docx]

**SUPPLEMENTAL MATERIALS**

**Differences observed in *Rickettsia parkeri* genes collected from three separate hard tick species (Family: Ixodidae)**

**TABLE OF CONTENTS**

**Supplemental Figure 1** 2

**Supplemental Figure 2** 3

**Supplemental Table 1** 4

**SUPPLEMENT 1).** EXPERIMENTAL METHODS 7

**SUPPLEMENT 2).** PCR AMPLICON REACTION MIXTURES AND CYCLE CONDITIONS 9

**SUPPLEMENT 3).** GENBANK ACCESSION NUMBERS 11

**SUPPLEMENT REFERENCES** 12

**
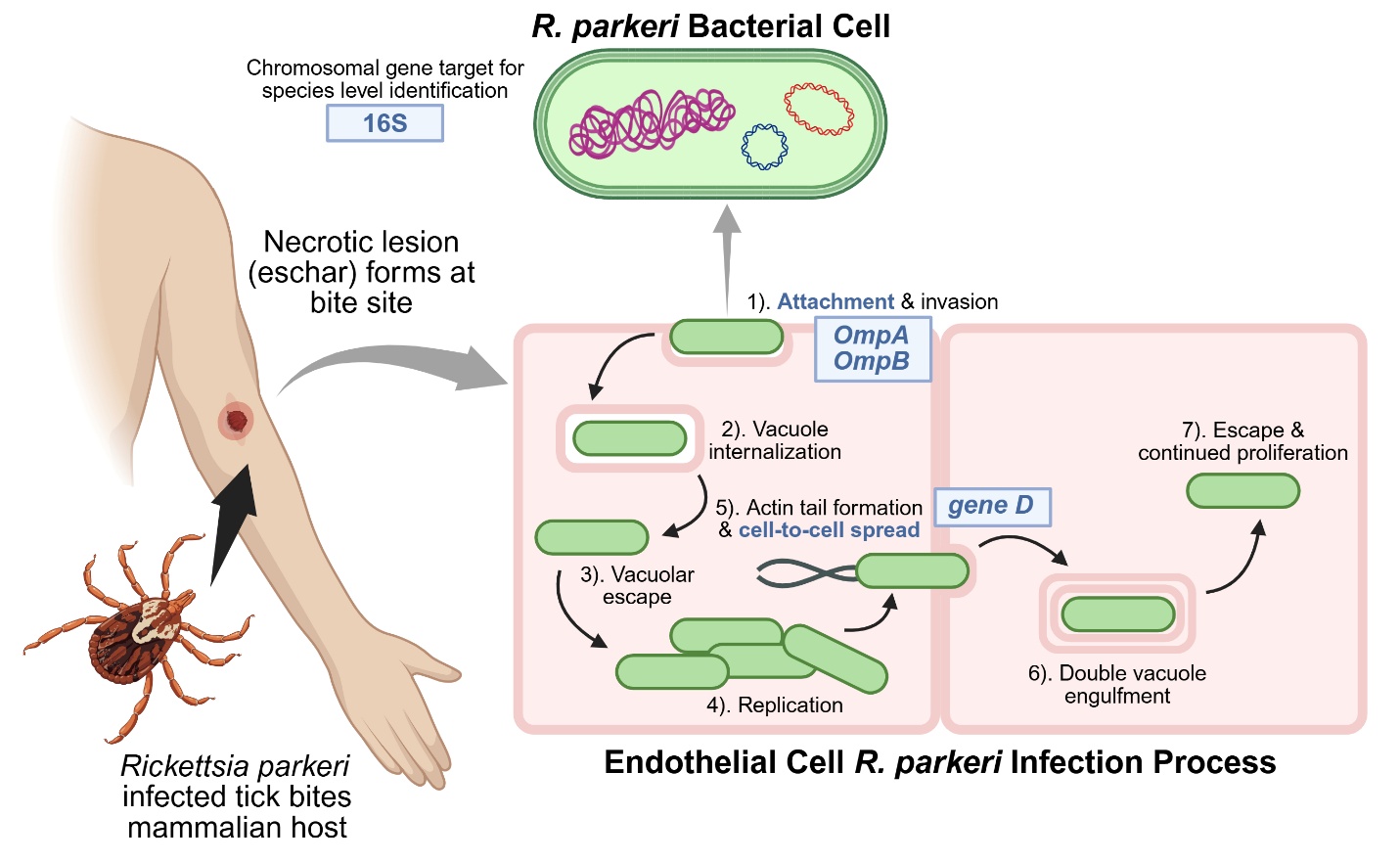
**

**Supplemental Figure 1.** A pictorial representation of the *Rickettsia parkeri* infection process in mammalian endothelial cells including the specific gene targets of interest for this study (16S, *OmpA*, *OmpB*, and *gene D*). *Image created in BioRender. Meyer, M. (2025)* [*https://BioRender.com/yhzscgh*](https://BioRender.com/yhzscgh)

*
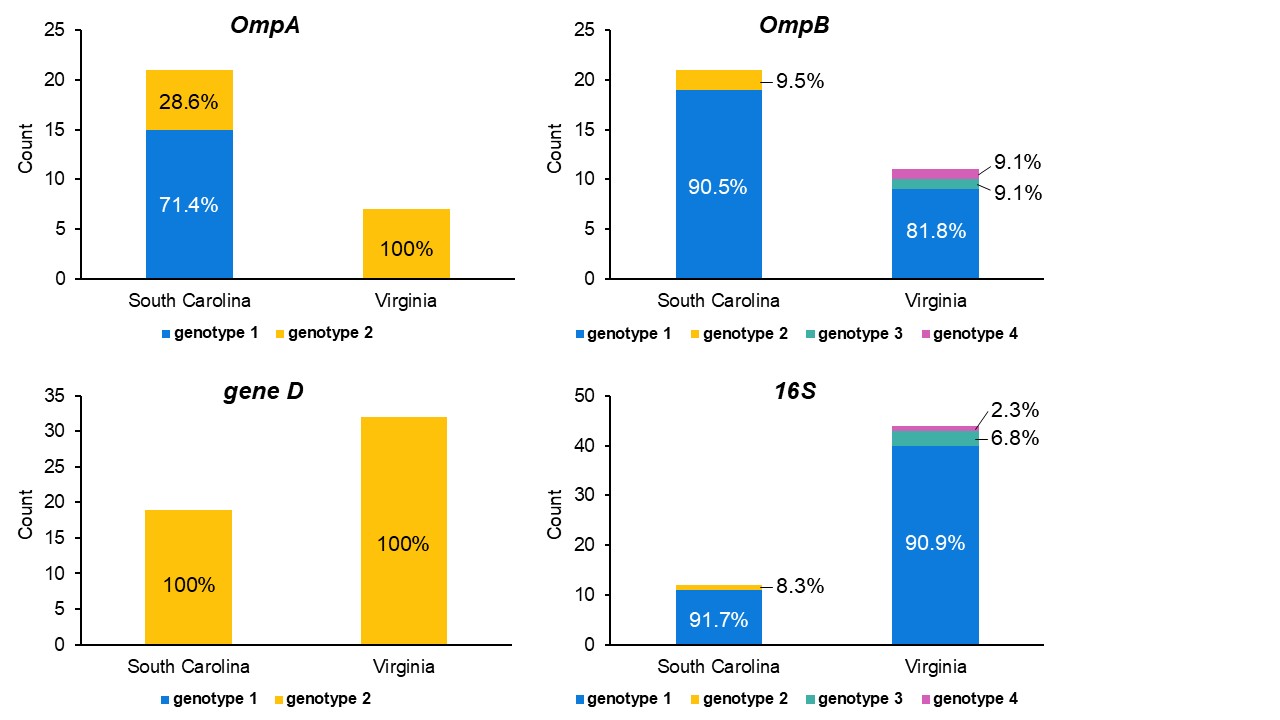
*

**Supplemental Figure 2.** Percentage of observed genotypes by geographic location, where the vertical axis denotes the number of tick samples (pooled or individual) per species analyzed

**Supplemental Table 1.** Collection details for all tick samples tested. Exact collection geographic location details were not provided for Virigina collections. Table rows are color coded by tick species.

| **Date** | **Tick ID** | **Species** | **Type** | **Sampling Method** | **Collection Location** | **Geographic Coordinates** |
| --- | --- | --- | --- | --- | --- | --- |
| 4/11/2023 | P23D0411-43-6-9 | *Amblyomma americanum* | Nymph | Carbon Dioxide Trap | Edisto Beach State Park, SC | (32.512785, -80.300377) |
| 4/11/2023 | P23D0411-39-10-12 | *Amblyomma americanum* | Male | Carbon Dioxide Trap | Edisto Beach State Park, SC | (32.512785, -80.300377) |
| 5/9/2023 | S23M40509-NP-21 | *Amblyomma maculatum* | Female | Animal Shelter | Lancaster Animal Shelter, SC | (34.7260398, -80.7351379) |
| 5/9/2023 | S23M40509-NP-30 | *Amblyomma maculatum* | Female | Animal Shelter | Lancaster Animal Shelter, SC | (34.7260398, -80.7351379) |
| 5/9/2023 | S23M40509-NP-37 | *Dermacentor variabilis* | Male | Animal Shelter | Lancaster Animal Shelter, SC | (34.7260398, -80.7351379) |
| 5/9/2023 | S23M40509-NP-33 | *Dermacentor variabilis* | Female | Animal Shelter | Lancaster Animal Shelter, SC | (34.7260398, -80.7351379) |
| 5/9/2023 | S23M40509-NP-59 | *Dermacentor variabilis* | Male | Animal Shelter | Lancaster Animal Shelter, SC | (34.7260398, -80.7351379) |
| 5/9/2023 | S23M40509-NP-35 | *Dermacentor variabilis* | Female | Animal Shelter | Lancaster Animal Shelter, SC | (34.7260398, -80.7351379) |
| 5/19/2023 | S23M40519-NP-124 | *Dermacentor variabilis* | Female | Animal Shelter | Lancaster Animal Shelter, SC | (34.7260398, -80.7351379) |
| 5/24/2023 | A1 | *Amblyomma americanum* | Female | Drag | Ivy Creek Park, VA | unspecified |
| 5/24/2023 | S23U70524-NP-135 | *Amblyomma maculatum* | Female | Animal Shelter | Palmetto Vet Med & Surgery McConnells, SC |  |
| 5/25/2023 | S23M40525-NP-136 | *Dermacentor variabilis* | Male | Animal Shelter | Lancaster Animal Shelter, SC | (34.7260398, -80.7351379) |
| 5/25/2023 | S23M40525-NP-137 | *Dermacentor variabilis* | Male | Animal Shelter | Lancaster Animal Shelter, SC | (34.7260398, -80.7351379) |
| 5/26/2023 | VDH-018 | *Amblyomma americanum* | Nymph | Drag | Gaines Farm, VA | unspecified |
| 5/26/2023 | VDH-022 | *Amblyomma americanum* | Nymph | Drag | Gaines Farm, VA | unspecified |
| 5/26/2023 | VDH-041 | *Amblyomma americanum* | Nymph | Drag | Gaines Farm, VA | unspecified |
| 5/26/2023 | A3 | *Amblyomma americanum* | Male | Drag | Gaines Farm, VA | unspecified |
| 5/26/2023 | S23M40526-NP-184 | *Amblyomma maculatum* | Female | Animal Shelter | Lancaster Animal Shelter, SC | (34.7260398, -80.7351379) |
| 5/26/2023 | S23M40526-NP-152 | *Amblyomma maculatum* | Male | Animal Shelter | Lancaster Animal Shelter, SC | (34.7260398, -80.7351379) |
| 5/26/2023 | S23M40526-NP-168 | *Dermacentor variabilis* | Female | Animal Shelter | Lancaster Animal Shelter, SC | (34.7260398, -80.7351379) |
| 5/26/2023 | S23M40526-NP-169 | *Dermacentor variabilis* | Male | Animal Shelter | Lancaster Animal Shelter, SC | (34.7260398, -80.7351379) |
| 5/26/2023 | S23M40526-NP-194 | *Dermacentor variabilis* | Female | Animal Shelter | Lancaster Animal Shelter, SC | (34.7260398, -80.7351379) |
| 5/26/2023 | S23M40526-NP-140 | *Dermacentor variabilis* | Male | Animal Shelter | Lancaster Animal Shelter, SC | (34.7260398, -80.7351379) |
| 5/26/2023 | S23M40526-NP-149 | *Dermacentor variabilis* | Female | Animal Shelter | Lancaster Animal Shelter, SC | (34.7260398, -80.7351379) |
| 5/26/2023 | S23M40526-NP-167 | *Dermacentor variabilis* | Female | Animal Shelter | Lancaster Animal Shelter, SC | (34.7260398, -80.7351379) |
| 6/2/2023 | A14 | *Amblyomma americanum* | Female | Drag | Lovers Lane, VA | unspecified |
| 6/6/2023 | VDH-098 | *Amblyomma americanum* | Nymph | Drag | Edge Hill Farm, VA | unspecified |
| 6/6/2023 | VDH-105 | *Amblyomma americanum* | Nymph | Drag | Edge Hill Farm, VA | unspecified |
| 6/6/2023 | VDH-109 | *Amblyomma americanum* | Nymph | Drag | Edge Hill Farm, VA | unspecified |
| 6/6/2023 | VDH-114 | *Amblyomma americanum* | Nymph | Drag | Edge Hill Farm, VA | unspecified |
| 6/15/2023 | VDH-128 | *Amblyomma americanum* | Nymph | Drag | Stoney Creek Park, VA | unspecified |
| 6/15/2023 | VDH-121 | *Amblyomma americanum* | Nymph | Drag | Mr. Scruby's Farm, VA | unspecified |
| 6/15/2023 | VDH-124 | *Amblyomma americanum* | Nymph | Drag | Mr. Scruby's Farm, VA | unspecified |
| 6/16/2023 | S23M40616-NP-260 | *Amblyomma maculatum* | Female | Animal Shelter | Lancaster Animal Shelter, SC | (34.7260398, -80.7351379) |
| 6/16/2023 | S23M40616-NP-227 | *Amblyomma maculatum* | Female | Animal Shelter | Lancaster Animal Shelter, SC | (34.7260398, -80.7351379) |
| 6/27/2023 | VDH-133 | *Amblyomma americanum* | Nymph | Drag | Sweetbriar College, VA | unspecified |
| 6/27/2023 | VDH-134 | *Amblyomma americanum* | Nymph | Drag | Sweetbriar College, VA | unspecified |
| 6/27/2023 | VDH-136 | *Amblyomma americanum* | Nymph | Drag | Sweetbriar College, VA | unspecified |
| 6/27/2023 | VDH-140 | *Amblyomma americanum* | Nymph | Drag | Sweetbriar College, VA | unspecified |
| 6/27/2023 | VDH-142 | *Amblyomma americanum* | Nymph | Drag | Sweetbriar College, VA | unspecified |
| 6/27/2023 | VDH-145 | *Amblyomma americanum* | Nymph | Drag | Sweetbriar College, VA | unspecified |
| 6/27/2023 | A27 | *Amblyomma americanum* | Female | Drag | Sweetbriar College, VA | unspecified |
| 6/27/2023 | A30 | *Amblyomma americanum* | Male | Drag | Sweetbriar College, VA | unspecified |
| 6/28/2023 | VDH-160 | *Amblyomma americanum* | Nymph | Drag | Pumpkin Creek Park, VA | unspecified |
| 6/28/2023 | VDH-161 | *Amblyomma americanum* | Nymph | Drag | Pumpkin Creek Park, VA | unspecified |
| 6/28/2023 | VDH-167 | *Amblyomma americanum* | Nymph | Drag | Peaks View Park, VA | unspecified |
| 6/28/2023 | VDH-169 | *Amblyomma americanum* | Nymph | Drag | Peaks View Park, VA | unspecified |
| 6/28/2023 | A38 | *Amblyomma americanum* | Male | Drag | Peaks View Park, VA | unspecified |
| 6/28/2023 | A35 | *Amblyomma americanum* | Male | Drag | Peaks View Park, VA | unspecified |
| 6/29/2023 | VDH-209 | *Amblyomma americanum* | Nymph | Drag | Liberty Lake Park, VA | unspecified |
| 6/29/2023 | VDH-210 | *Amblyomma americanum* | Nymph | Drag | Smith Mountain Lake State Park, VA | unspecified |
| 6/29/2023 | VDH-211 | *Amblyomma americanum* | Nymph | Drag | Smith Mountain Lake State Park, VA | unspecified |
| 6/29/2023 | VDH-213 | *Amblyomma americanum* | Nymph | Drag | Smith Mountain Lake State Park, VA | unspecified |
| 6/29/2023 | VDH-217 | *Amblyomma americanum* | Nymph | Drag | Smith Mountain Lake State Park, VA | unspecified |
| 6/29/2023 | VDH-218 | *Amblyomma americanum* | Nymph | Drag | Smith Mountain Lake State Park, VA | unspecified |
| 6/29/2023 | VDH-222 | *Amblyomma americanum* | Nymph | Drag | Smith Mountain Lake State Park, VA | unspecified |
| 6/29/2023 | VDH-224 | *Amblyomma americanum* | Nymph | Drag | Smith Mountain Lake State Park, VA | unspecified |
| 6/29/2023 | VDH-225 | *Amblyomma americanum* | Nymph | Drag | Smith Mountain Lake State Park, VA | unspecified |
| 6/29/2023 | VDH-226 | *Amblyomma americanum* | Nymph | Drag | Smith Mountain Lake State Park, VA | unspecified |
| 6/29/2023 | VDH-230 | *Amblyomma americanum* | Nymph | Drag | Smith Mountain Lake State Park, VA | unspecified |
| 6/29/2023 | VDH-234 | *Amblyomma americanum* | Nymph | Drag | Smith Mountain Lake State Park, VA | unspecified |
| 6/29/2023 | VDH-237 | *Amblyomma americanum* | Nymph | Drag | Smith Mountain Lake State Park, VA | unspecified |
| 6/29/2023 | VDH-240 | *Amblyomma americanum* | Nymph | Drag | Smith Mountain Lake State Park, VA | unspecified |
| 6/29/2023 | A43 | *Amblyomma americanum* | Female | Drag | Liberty Lake Park, VA | unspecified |
| 6/29/2023 | VDH-208 | *Amblyomma americanum* | Nymph | Drag | Liberty Lake Park, VA | unspecified |
| 6/29/2023 | VDH-216 | *Amblyomma americanum* | Nymph | Drag | Smith Mountain Lake State Park, VA | unspecified |
| 6/29/2023 | VDH-221 | *Amblyomma americanum* | Nymph | Drag | Smith Mountain Lake State Park, VA | unspecified |
| 6/29/2023 | VDH-229 | *Amblyomma americanum* | Nymph | Drag | Smith Mountain Lake State Park, VA | unspecified |
| 6/29/2023 | VDH-241 | *Amblyomma americanum* | Nymph | Drag | Smith Mountain Lake State Park, VA | unspecified |
| 7/3/2023 | S23M40703-NP-293 | *Dermacentor variabilis* | Female | Animal Shelter | Lancaster Animal Shelter, SC | (34.7260398, -80.7351379) |
| 7/11/2023 | VDH-243 | *Amblyomma americanum* | Nymph | Drag | Roanoke, VA | unspecified |
| 7/11/2023 | VDH-244 | *Amblyomma americanum* | Nymph | Drag | Roanoke, VA | unspecified |
| 7/11/2023 | VDH-251 | *Amblyomma americanum* | Nymph | Drag | Roanoke, VA | unspecified |
| 7/11/2023 | VDH-253 | *Amblyomma americanum* | Nymph | Drag | Roanoke, VA | unspecified |
| 7/28/2023 | S23M40728-NP-322 | *Dermacentor variabilis* | Female | Animal Shelter | Lancaster Animal Shelter, SC | (34.7260398, -80.7351379) |
| 7/28/2023 | S23M40728-NP-323 | *Dermacentor variabilis* | Female | Animal Shelter | Lancaster Animal Shelter, SC | (34.7260398, -80.7351379) |
| 7/28/2023 | S23M40728-NP-324 | *Dermacentor variabilis* | Male | Animal Shelter | Lancaster Animal Shelter, SC | (34.7260398, -80.7351379) |
| 8/11/2023 | S23M40811-NP-358 | *Dermacentor variabilis* | Male | Animal Shelter | Lancaster Animal Shelter, SC | (34.7260398, -80.7351379) |
| 6/27/2025 | VDH-152 | *Amblyomma americanum* | Nymph | Drag | Sweetbriar College, VA | unspecified |

**SUPPLEMENT 1).** EXPERIMENTAL METHODS

**Tick Collection Methods**

Questing ticks were collected via CO_2_ traps and dragging methods based on the US Centers for Disease Control and Prevention guidelines throughout the year of 2023 [1, 2]. Tick drags were constructed from 1.22 x 1.52 m white duck canvas attached to a 1.22 m wooden dowel with zinc washers attached as weights to the bottom of the canvas. Dragging was conducted in 30-minute increments along natural regions of interest with checks every 30 seconds for ticks. Any ticks attached to the drag cloth were removed and placed in 75% ethanol for storage. Passive traps baited with CO_2_ contained 0.61 m^2^ white muslin squares with 0.5-1kg of dry ice placed in the center of each muslin square. Traps were placed in leaf litter or grass with 10 traps placed per collection location. Traps were left undisturbed for 1.5-2 hours before removal. Ticks were removed from the traps and again stored in 75% ethanol. Nine animal shelters across the state of South Carolina collected ticks from stray dogs brought to their facilities throughout 2023. Ticks were removed from the dogs and placed in 15 mL of 75% ethanol. Collection details for all tick samples are provided in Supplemental Table 2.

**DNA Purification and Sample Information**

Ticks were bisected longitudinally, with half the tick going to analysis and the other half banked for long-term storage. Questing tick halves were pooled by species, sex, life stage, collection method, collection date, and location with the following maximum pool sizes: a single pool for all larvae, pool of five for nymphs, and pool of three for adults. Host-attached ticks were bisected and tested individually. All samples were then bead homogenized in 180 µL Qiagen Buffer ATL Germantown, MD, USA) using the Qiagen TissueLyser II (Germantown, MD, USA) and 5 mm stainless steel beads after which 20 µL of Proteinase K was added to each sample. Tick homogenate was then incubated overnight at 56ºC. Following incubation, samples were allowed to return to room temperature and 150 µL of tick lysate was extracted using the Qiagen QIAmp 96 DNA QIAcube HT kit (Germantown, MD, USA) and the Qiagen QIAcube HT robotic workstation (Germantown, MD, USA). The final purified nucleic acid extracts were eluted in 100 µL Qiagen AE (Germantown, MD, USA). Nucleic acid yield and quality was validated using the Qiagen QIAxpert System (Germantown, MD, USA). Polymerase chain reaction (RT-PCR) pathogen testing methods can be found in our previous publication [3].

**PCR Amplicon Preparation**

PCR amplification was conducted for the four *Rickettsia parkeri* gene targets used in this study. Detailed reaction mixtures and cycle conditions are supplied in Supplement 2. All PCR amplifications were conducted on an Applied Biosystems QuantStudio 5 Real-Time PCR System (Thermo Fisher Scientific, Waltham, MA). Amplification was confirmed using an Agilent TapeStation and the D1000 or D5000 ScreenTape Assay (Santa Clara, CA). Amplicons were purified using an AMPure XP Bead (Beckman Coulter, Brea, CA) cleanup. Final DNA concentrations were measured using the Qiagen QIAxpert (Hilden, Germany).

**Nanopore Sequencing & Data Analysis**

Following target amplification and purification, samples were prepared for long-read sequencing using Oxford Nanopore Technology’s (ONT) Native Barcoding Kit (Oxford, United Kingdom) and sequenced using an ONT MinIon Mk1c (Oxford, United Kingdom).

Consensus sequences were generated using the ONT EPI2ME platform using the Amplicon workflow and the following NCBI GenBank references:

- *OmpA* – Accession Number KY271186.1
- *OmpB*  – Accession Number AF123717.1
- *gene D*  – Accession Number KY113112.1
- *16S*  – Accession Number NR_029156.1

Read lengths were filtered to include only the relevant amplicon size range and no filtered reads were down sampled for analysis. Minimum mean read quality was set to 10. Final consensus sequences and references were aligned references using MUSCLE and phylogenetic trees were created using MEGA11 with rooting on the *Rickettsia felis* outgroup [4, 5, 6]. The optimal evolutionary models were identified using MEGA11 [5]. Evolutionary histories for Figure 1 trees were inferred using the Maximum Likelihood method and the Hasegawa-Kishino-Yano mode with a uniform distribution for *OmpA* and a discrete Gamma distribution for *OmpB* [5, 7]. Both trees were inferred using 500 replicates and branches reproduced in less than 50% of bootsrap replicates were collapsed. The final evolutionary history for Figure 2 was inferred using the Maximum Likelihood method and General Time Reversible model with a discrete Gamma distribution and 500 replicates [5, 7]. Branches reproduced in less than 70% of bootstrap replicates were collapsed.

**SUPPLEMENT 2).** PCR AMPLICON REACTION MIXTURES AND CYCLE CONDITIONS

**R. parkeri amplicon sequencing primers by gene:**

- **sca0 (aka, OmpA);** 532 bp:
- P1 (Rr190.70p): ATG GCG AAT ATT TCT CCA AAA
- P3 (Rr190.602n): AGT GCA GCA TTC GCT CCC CCT
- **sca4 (aka, gene D);** 2,302 bp:
- P1: CGA TGG TAG CAT TAA AAG CT
- P2: TCA GCG TTG TGG AGG GGA AG
- **sca5 (aka, OmpB)**; 2,929 bp:
- P1 (120-M59): CCG CAG GGT TGG TAA CTG C
- P2 (120-2988): CCG GCT ATA CCG CCT GTA GT
- **rrs (aka, 16S)**; 1,462 bp:
- P1: AGA GTT TGA TCC TGG CTC AG
- P2: ACG GCT ACC TTG TTA CGA CTT

**16S/rrs (~1,462 bp amplicon)**

**Volume per reaction (25 µL total reaction volume)**

- 12.5 µL Thermo Scientific DreamTaq Buffer (10X)
- 1.25 µL 10 µM primer 1 (final concentration 0.5 µM)
- 1.25 µL 10 µM primer 2 (final concentration 0.5 µM)
- 7.5 µL nuclease free water
- 2.5 µL template DNA

**Cycle Conditions**

3 min at 95°C

35 cycles of:

30 sec at 95°C

30 sec at 42°C

1 min at 72°C

15 min at 72°C

**Lit references:** [6, 8]

**sca4/gene D (~2,302 bp amplicon)**

**Volume per reaction (25 µL total reaction volume)**

- 12.5 µL Thermo Scientific DreamTaq Buffer (10X)
- 0.5 µL 10 µM primer 1 (final concentration 0.2 µM)
- 0.5 µL 10 µM primer 2 (final concentration 0.2 µM)
- 9.0 µL nuclease free water
- 2.5 µL template DNA

**Cycle Conditions**

3 min at 95°C

35 cycles of:

30 sec at 95°C

30 sec at 50°C

1 min at 72°C

5 min at 72°C

**Lit references:** [6, 9]

**OmpB/sca5 (~2,929 bp amplicon)**

**Volume per reaction (25 µL total reaction volume)**

- 12.5 µL New England Biolabs LongAmp Taq 2X Master Mix
- 1.0 µL 10 µM primer 1 (final concentration 0.4 µM)
- 1.0 µL 10 µM primer 2 (final concentration 0.4 µM)
- 8.0 µL nuclease free water
- 2.5 µL template DNA

**Cycle Conditions**

3 min at 94°C

35 cycles of:

30 sec at 94°C

30 sec at 50°C

2 min and 30 sec at 65°C

10 min at 65°C

**Lit references:** [6, 10]

**OmpA/sca0 (~532 bp amplicon)**

**Volume per reaction (20 µL total reaction volume)**

- 10 µL Quantbio PerfCTa qPCR ToughMix Low ROX
- 1.0 µL 10 µM primer 1 (final concentration 1.25 µM)
- 1.0 µL 10 µM primer 2 (final concentration 1.25 µM)
- 6.0 µL nuclease free water
- 2.0 µL template DNA

**Cycle Conditions**

3 min at 95°C

35 cycles of:

20 sec at 95°C

30 sec at 46°C

1 min sec at 60°C

60 min at 60°C

**Lit references:** [6, 11, 12]

**SUPPLEMENT 3).** GENBANK ACCESSION NUMBERS

**OmpA**

29 sequences were submitted to GenBank with accession numbers:

PV877555 – PV877583

**OmpB**

33 sequences were submitted to GenBank with accession numbers:

PV877665 – PV877666 and PV920701-PV920702.

**gene D**

52 sequences were submitted to GenBank with accession numbers:

PV877584 – PV877635

**16S**

57 sequences were submitted to GenBank with accession numbers:

PV854078 – PV854134

**SUPPLEMENT REFERENCES**

1. CDC: Surveillance for Ixodes scapularis and pathogens found in this tick species in the United States. Edited by DVBD2019.

2. CDC: Guide to the Surveillance of Metastriate Ticks (Acari: Ixodidae) and their Pathogens in the United States. Edited by DVBD2020.

3. Gual-Gonzalez L, Self SCW, Zellars K, Meyer M, Dye-Braumuller KC, Evans CL, et al. Eco-epidemiology of *Rickettsia amblyommatis* and *Rickettsia parkeri* in naturally infected ticks (Acari: Ixodida) from South Carolina. Parasites & Vectors. 2024;17 1:33; doi: 10.1186/s13071-023-06099-z. <https://doi.org/10.1186/s13071-023-06099-z>.

4. Edgar RC. MUSCLE: a multiple sequence alignment method with reduced time and space complexity. BMC Bioinformatics. 2004;5 1:113; doi: 10.1186/1471-2105-5-113. <https://doi.org/10.1186/1471-2105-5-113>.

5. Tamura K, Stecher G, Kumar S. MEGA11: Molecular Evolutionary Genetics Analysis Version 11. Molecular Biology and Evolution. 2021;38 7:3022-7; doi: 10.1093/molbev/msab120. <https://doi.org/10.1093/molbev/msab120>.

6. Paddock CD, Allerdice MEJ, Karpathy SE, Nicholson WL, Levin ML, Smith TC, et al. Unique Strain of *Rickettsia parkeri* Associated with the Hard Tick *Dermacentor parumapertus* Neumann in the Western United States. Applied and Environmental Microbiology. 2017;83 9:e03463-16; doi: doi:10.1128/AEM.03463-16. <https://journals.asm.org/doi/abs/10.1128/aem.03463-16>.

7. Hasegawa M, Kishino H, Yano T. Dating of the human-ape splitting by a molecular clock of mitochondrial DNA. J Mol Evol. 1985;22 2:160-74; doi: 10.1007/bf02101694.

8. Weisburg WG, Barns SM, Pelletier DA, Lane DJ. 16S ribosomal DNA amplification for phylogenetic study. Journal of Bacteriology. 1991;173 2:697-703; doi: doi:10.1128/jb.173.2.697-703.1991. <https://journals.asm.org/doi/abs/10.1128/jb.173.2.697-703.1991>.

9. Sekeyova Z, Roux V, Raoult D. Phylogeny of *Rickettsia* spp. inferred by comparing sequences of 'gene D', which encodes an intracytoplasmic protein. International Journal of Systematic and Evolutionary Microbiology. 2001;51 4:1353-60; doi: <https://doi.org/10.1099/00207713-51-4-1353>. <https://www.microbiologyresearch.org/content/journal/ijsem/10.1099/00207713-51-4-1353>.

10. Roux V, Raoult D. Phylogenetic analysis of members of the genus *Rickettsia* using the gene encoding the outer-membrane protein rOmpB (ompB). International Journal of Systematic and Evolutionary Microbiology. 2000;50 4:1449-55; doi: <https://doi.org/10.1099/00207713-50-4-1449>. <https://www.microbiologyresearch.org/content/journal/ijsem/10.1099/00207713-50-4-1449>.

11. Regnery RL, Spruill CL, Plikaytis BD. Genotypic identification of rickettsiae and estimation of intraspecies sequence divergence for portions of two rickettsial genes. Journal of Bacteriology. 1991;173 5:1576-89; doi: doi:10.1128/jb.173.5.1576-1589.1991. <https://journals.asm.org/doi/abs/10.1128/jb.173.5.1576-1589.1991>.

12. Roux V, Fournier PE, Raoult D. Differentiation of spotted fever group rickettsiae by sequencing and analysis of restriction fragment length polymorphism of PCR-amplified DNA of the gene encoding the protein rOmpA. Journal of Clinical Microbiology. 1996;34 9:2058-65; doi: doi:10.1128/jcm.34.9.2058-2065.1996. <https://journals.asm.org/doi/abs/10.1128/jcm.34.9.2058-2065.1996>.
